# Impact of bromocriptine withdrawal timing on pregnancy outcomes in patients with pituitary prolactin-secreting microadenomas: a real-world retrospective study of 188 pregnancies in China

**DOI:** 10.3389/fphar.2026.1806870

**Published:** 2026-04-02

**Authors:** Jiao Lan, Peng Hong, Wei Huang, Aiyun Xing, Peizhi Zhou, Jing Tan, Xue Peng, Jing Fu

**Affiliations:** 1 Department of Obstetrics and Gynaecology, West China Second University Hospital, Sichuan University, Chengdu, Sichuan, China; 2 Department of Obstetrics and Gynaecology, The First People’s Hospital of Shuangliu District, Chengdu, Sichuan, China; 3 Department of Obstetrics and Gynaecology, Qamdo People’s Hospital of Tibet Autonomous Region, Qamdo, China; 4 Key Laboratory of Birth Defects and Related Diseases of Women and Children, Ministry of Education, Chengdu, Sichuan, China; 5 Department of Neurosurgery, West China Hospital/West China School of Medicine, Sichuan University, Chengdu, Sichuan, China

**Keywords:** bromocriptine, dopamine agonist therapy, fetal development, pituitary prolactin-secreting microadenoma, pregnancy outcomes

## Abstract

**Background:**

This study aims to evaluate the influence of the timing for bromocriptine withdrawal in pregnant women with pituitary prolactin-secreting microadenomas.

**Methods:**

In this retrospective study,188 pregnant women diagnosed with pituitary prolactin-secreting microadenomas, who delivered between January 2005 and June 2023 were categorized into four groups based on their use and duration of bromocriptine therapy. Their pregnancy and neonatal outcomes were analyzed.

**Results:**

Among the 188 patients, the incidence of neurological symptoms during pre-pregnancy varied significantly across the groups (P = 0.000), with the highest prevalence in Group A (21.7%) and minimal to no symptoms in other Groups. The live birth rates and miscarriage rates did not show statistically significant differences across the groups (P = 0.508). Premature birth rates were 6.5%, 6.2%, 13.2%, and 0% for Groups A, B, C, and D, respectively. Notably, the incidence of small-for-gestational-age (SGA) infants increased with prolonged bromocriptine use, while large-for-gestational-age (LGA) rates decreased, though these trends were not statistically significant (P = 0.068). Median gestational age differed significantly across groups (P = 0.000), but neonatal weights remained comparable (P = 0.471).

**Conclusion:**

Discontinuation of bromocriptine after pregnancy confirmation is generally safe for patients with pituitary prolactin-secreting microadenomas. However, for patients with pre-existing neurological or ophthalmological symptoms, or those untreated prior to pregnancy, close monitoring and potential continuation of dopamine agonist therapy are waranted. Bromocriptine exposure throughout pregnancy does not increase the risk of miscarriage, congenital anomalies, or adverse neonatal outcomes. But further prospective studies are needed to elucidate the long-term effects of bromocriptine on fetal development.

## 1 Introduction

Pituitary prolactinomas, accounting for approximately 50% of all pituitary tumors, are the most common functional neuroendocrine tumors (Chanson and Maiter, 2019). These lesions are characterized by excess prolactin secretion and are categorized as macroadenomas (≥1 cm) or microadenomas (<1 cm), with the latter being more prevalent in women of reproductive age (Molitch, 2015). Clinically, prolactinomas in women typically present with amenorrhea or oligomenorrhea in 85%–90% of cases, alongside symptoms such as galactorrhea and infertility, while a smaller proportion of patients experience neurological manifestations, including headaches and visual disturbances (Lamba et al., 2019). Prolactin secretion is primarily regulated by hypothalamic dopamine, and thus, dopamine agonists (DAs) like bromocriptine and cabergoline are the cornerstone of treatment, effectively reducing prolactin levels, inducing tumor shrinkage, and restoring reproductive function (Melmed et al., 2011; Wong et al., 2015).

Given the high prevalence of prolactinomas in women of childbearing age, approximately 85% of women receiving DA therapy express a desire for pregnancy. Although the use of bromocriptine early in pregnancy has not been shown to significantly increase the risks of miscarriage, preterm birth, or congenital anomalies (Krupp and Monka, 1987; Barraud et al., 2020; Ruiz-Velasco and Tolis, 1984), international consensus guidelines recommend discontinuing DAs upon confirmation of pregnancy to limit fetal exposure (Petersenn et al., 2023). Nevertheless, studies have reported that a significant proportion of women continue DA therapy beyond pregnancy confirmation without apparent harm to the fetus. For example, in a cohort of 1,414 hyperprolactinemic women, 90% continued bromocriptine therapy for more than 15 days after pregnancy was confirmed, with no significant differences in fetal outcomes observed (Ruiz-Velasco and Tolis, 1984).

The question of whether the duration of bromocriptine therapy during pregnancy affects maternal and fetal outcomes remains unresolved. Existing guidelines offer limited advice on managing prolactin-secreting microadenomas in pregnant patients, particularly regarding optimal drug cessation timing. This study addresses this gap by retrospectively analyzing clinical outcomes in a cohort of pregnant women with prolactin-secreting microadenomas, stratified by the duration of bromocriptine therapy. Our goal is to provide evidence-based insights into the management of prolactinomas during pregnancy, aiding in clinical decision-making for this patient population.

## 2 Materials and methods

### 2.1 Data source

**FIGURE 1:**
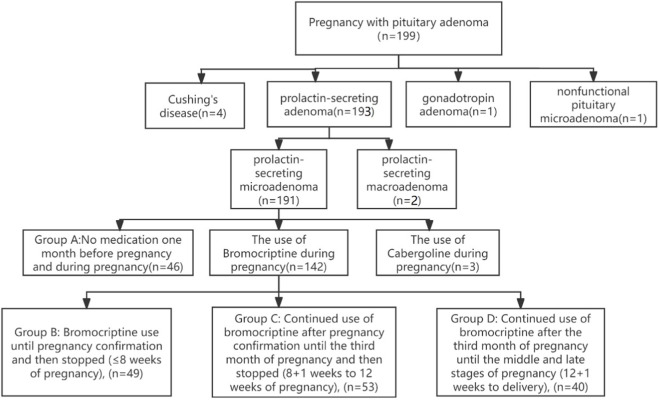
Flowchart of patient inclusion and grouping by bromocriptine use during pregnancy.

This retrospective study was conducted at the Department of Obstetrics and gynecology, West China Second University Hospital of Sichuan University. Data were obtained from the hospital’s information system, covering cases delivered after 12 weeks between January 2006 and May 2023. A total of 199 pregnancy women with pituitary adenoma were initially identified. Following the application of inclusion and exclusion criteria, 191 cases of prolactin-secreting microadenomas were identified as the study population. Of these, three patients were treated with cabergoline and excluded from the analysis, resulting in 188 patients included in the final analysis Figure 1.

### 2.2 Study population

The inclusion criteria for this study were women diagnosed with prolactin-secreting microadenomas between January 2006 and May 2023, underwent regular prenatal follow-ups, and delivered after 12 weeks at West China Second University Hospital of Sichuan University. Diagnosis of prolactinoma:Clinical presentation: Hyperprolactinaemia and hypogonadism; with serum prolactin levels exceeding the upper limit of normal values, and imaging showing a mass in the sella turcica. Which conforms to the Endocrine Society Clinical Practice Guidelines (Melmed et al., 2011) and the more recent Pituitary Society International Consensus Statement (Petersenn et al., 2023).

Exclusion criteria included patients with growth hormone adenomas, acromegaly, Cushing’s disease, thyrotropin adenomas, adrenocorticotropic hormone adenomas, and nonfunctional pituitary adenoma, or women with prolactin-secreting microadenomas using cabergoline. Informed consents were obtained and ethical approval for the study was obtained from the Medical Research Ethics Committee of West China Second University Hospital of Sichuan University (Approval No. 2024078).

### 2.3 Study design and grouping

The exact cumulative pre-pregnancy dose and duration varied significantly as many patients were managed in external clinics before referral,so patients were categorized into four groups just based on the duration and timing of bromocriptine therapy relative to pregnancy:

Group A: Patients who discontinued bromocriptine 1 month prior to pregnancy and did not receive any treatment during pregnancy (n = 46).

Group B: Patients who discontinued bromocriptine upon confirmation of pregnancy (up to 8 weeks gestation) (n = 49).

Group C: Patients who continued bromocriptine therapy until the end of the first trimester (8 + 1–12 weeks gestation) (n = 53).

Group D: Patients who continued bromocriptine therapy beyond the first trimester and into the second or third trimesters (from 12+1 week until delivery) (n = 40).

### 2.4 Outcome measures

The primary outcomes assessed in this study were pregnancy and neonatal outcomes, including live birth rates, miscarriage rates, and neonatal gestational age at birth. Secondary outcomes included the incidence of pregnancy complications such as gestational diabetes, pregnancy-induced hypertension, and delivery methods. Additionally, the incidence of small-for-gestational-age (SGA) and large-for-gestational-age (LGA) infants, as well as birth weights, were recorded for comparison across the four groups.

### 2.5 Statistical analysis

Statistical analyses were performed using SPSS 20.0 software. Continuous variables following a normal distribution were reported as mean ± standard deviation (SD), while non-normally distributed variables were expressed as medians with interquartile ranges (IQR). One-way analysis of variance (ANOVA) or the Kruskal–Wallis H test was used for between-group comparisons of continuous variables. Categorical variables were expressed as frequencies and percentages, and the chi-square test or Fisher’s exact test was employed for comparisons between groups. A p-value of less than 0.05 was considered statistically significant.

## 3 Results

Due to the long-term retrospective nature of the cohort, systematic data on pre-gestational chronic hypertension and diabetes were not uniformly available in the electronic records for all patients. Comorbidities were not analyzed.

### 3.1 General clinical characteristics of 188 pregnant patients with prolactin-secreting microadenomas

The clinical characteristics of the patients prior to pregnancy varied significantly between the four groups (P = 0.000), particularly in terms of neurological symptoms. Group A exhibited the highest prevalence of neurological symptoms before pregnancy, with 21.7% of patients (10/46) reporting such symptoms, while none were observed in Groups C and D. Additionally, the rate of adverse obstetric history was significantly different between the groups (P = 0.015), with Group D displaying the highest proportion of patients with two or more previous adverse outcomes (17.5%, 7/40). The method of conception also differed significantly (P = 0.012), with Group D showing a lower rate of natural conception (75%, 30/40) compared to the other groups. However, no significant differences were observed in terms of age, ethnicity, or menstrual cycle regularity among the groups. The detailed clinical characteristics of each group are presented in Table1.

**TABLE 1:** Comparison of pre-pregnancy clinical characteristics across the four patient groups.

Baseline characteristic	​	Group A (n = 46)	Group B (n = 49)	Group C (n = 53)	Group D (n = 40)	P
Age at pregnancy (years)	​	30.5 (27.75–35.0)	29 (27–32)	30 (29–34)	31 (29–34.75)	0.092
Regularity of the menstrual cycle (%)	Regular	30 (65.2)	30 (61.2)	43 (81.1)	31 (77.5)	0.9
	Irregular	16 (34.8)	19 (38.8)	10 (18.9)	9 (22.5)	
Clinical symptoms (%)	Endocrine symptoms	17 (37.0)	39 (79.6)	33 (62.2)	27 (67.5)	0.000
	Neurological symptoms	10 (21.7)	1 (2.04)	0	0	
	None	2 (4.3)	1 (2.04)	10 (18.9)	2 (5)	
	Unknown	17 (37.0)	8 (16.3)	10 (18.9)	11 (27.5)	
Mode of conception (%)	Natural conception	45 (97.8)	42 (85.7)	44 (83.0)	30 (75)	0.012
	Assisted reproduction	1 (2.2)	7 (14.3)	9 (17.0)	10 (25)	
Adverse obstetric history (%)	None	37 (80.4)	45 (91.8)	38 (71.7)	24 (60.0)	0.015
	One time	6 (13.0)	4 (8.2)	10 (18.9)	9 (22.5)	
	Two times	2 (4.3)	0	5 (9.4)	6 (15.0)	
	Three or more times	1 (2.3)	0	0	1 (2.5)	
Ethnicity (%)	Han ethnicity	46 (100)	49 (100)	51 (96.2)	40 (100)	0.249
	Ethnic minorities	0	0	2 (3.8)	0	

### 3.2 Pregnancy outcomes in patients with prolactin-secreting microadenomas

The live birth rate, miscarriage rate, and stillbirth rate across the four groups were not statistically significant (P = 0.508). Three cases of fetal malformations were observed, one in each of Groups A, B, and C. These included one case of trisomy 21 and trisomy 18 in Group C, one case of fetal edema in Group B, and one case of stillbirth in Group A, all resulting in pregnancy termination before 28 weeks gestation. Two patients in Group A experienced worsening headaches and significant vision loss during pregnancy, necessitating pregnancy termination followed by neurosurgical intervention. Additionally, the mode of delivery varied significantly between the groups (P = 0.017), with Group D showing the highest rate of cesarean section (77.5%, 32/40) and Group B the lowest (46.9%, 23/49). The incidence of gestational diabetes and pregnancy-induced hypertension did not differ significantly across the groups, with P-values of 0.945 and 0.878, respectively. Detailed pregnancy outcomes are shown in Table 2.

**TABLE 2:** Pregnancy outcomes in patients with prolactin-secreting microadenomas.

Pregnancy outcomes	​	Group A (n = 46)	Group B (n = 49)	Group C (n = 53)	Group D (n = 40)	P
Gestational diabetes (%)	Yes	15 (32.6)	14 (28.6)	18 (34)	12 (30)	0.945
	No	31 (67.4)	35 (71.4)	35 (66)	28 (70)	
Pregnancy-induced hypertension (%)	Yes	1 (2.2)	2 (4.1)	3 (5.7)	1 (2.5)	0.878
	No	45 (97.8)	47 (95.9)	50 (94.3)	39 (97.5)	
Mode of delivery (%)	Vaginal delivery Cesarean section	18 (39.1) 28 (60.9)	26 (53.1) 23 (46.9)	16 (30.2) 37 (69.8)	9 (22.5) 31 (77.5)	0.017
Pregnancy outcomes (%)	Live birth	43 (93.5)	48 (98)	52 (98.1)	40 (100)	0.508
	Miscarriage	1 (2.2)	1 (2)	1 (1.9)	0	
	Stillbirth	2 (4.3)	0	0	0	

### 3.3 Neonatal outcomes in patients with prolactin-secreting microadenomas

**FIGURE 2:**
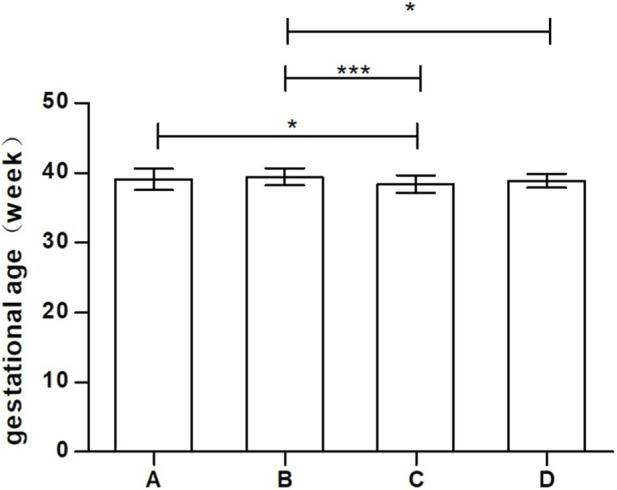
Distribution of Neonatal Gestational Age across the four patient groups.

The rate of multiple pregnancies was 5.32% (10/188), with no statistically significant differences between the groups (P = 0.465). Premature birth rates were 6.5% (3/46), 6.2% (3/49), 13.2% (7/53), and 0% in Groups A, B, C, and D, respectively. The incidence of small-for-gestational-age (SGA) infants was 0% in Groups A and B, 3.8% (2/53) in Group C, and 5% (2/40) in Group D. In contrast, the incidence of large-for-gestational-age (LGA) infants decreased with prolonged bromocriptine use: 6.5% (3/46) in Group A, 2% (1/49) in Group B, 1.9% (1/53) in Group C, and 0% in Group D. The miscarriage rates were 6.5% (3/46), 2% (1/49), 1.9% (1/53), and 0% in Groups A, B, C, and D, respectively. While these differences were not statistically significant (P = 0.068), a trend was observed with increased SGA incidence and decreased LGA and miscarriage incidence as bromocriptine use continued into later pregnancy. No significant differences were found in neonatal birth weights (P = 0.471) or Apgar scores across the groups (P = 1.00). Additionally, only one case of neonatal syndactyly was reported in Group B. The gestational age of neonates differed significantly between the groups (P = 0.000), with Group B showing the longest median gestational age (39.43 weeks). Detailed neonatal outcomes are presented in Table 3 and Figure 2.

**TABLE 3:** Neonatal outcomes in patients with prolactin-secreting microadenomas by bromocriptine use duration.

Neonatal outcomes	​	Group A (n = 46)	Group B (n = 49)	Group C (n = 53)	Group D (n = 40)	P
Number of fetuses (%)	Singleton	43 (93.5)	48 (98)	48 (94.3)	37 (92.5)	0.465
	Multiple fetuses	3 (6.5)	1 (2)	3 (5.7)	3 (7.5)	
Neonatal outcomes (%)	Term healthy infant	37 (80.4)	44 (89.8)	42 (79.2)	38 (95)	0.068
	Preterm infant	3 (6.5)	3 (6.2)	7 (13.2)	0	
	SGA	0	0	2 (3.8)	2 (5)	
	LGA	3 (6.5)	1 (2)	1 (1.9)	0	
	Miscarried infant	3 (6.5)	1 (2)	1 (1.9)	0	
Apgar score 8–10 (%)	1min	46	49	53	40	1
	5min	46	49	53	40	
	10min	46	49	53	40	
The birth weight of neonates (kg)		3.20 ± 0.51	3.29 ± 0.40	3.16 ± 0.42	3.16 ± 0.38	0.471
Gestational age of neonates (week)		39.29 (38.71–40)	39.43 (39.14–0.11)	38.71 (37.61–39.25)	39 (38–39.68)	0.000

For twins or multiple fetuses, take the average value of neonatal weight.

## 4 Discussion

The management of prolactin-secreting microadenomas in pregnant women, particularly regarding the timing of dopamine agonist (DA) withdrawal, remains a clinical challenge. In this study, we retrospectively analyzed 188 pregnancies complicated by prolactinomas to assess the impact of bromocriptine withdrawal timing on both maternal and fetal outcomes. Our findings contribute to the understanding of optimal management strategies for these patients, especially those who continue DA therapy during pregnancy.

Firstly, our results indicate that patients who discontinued bromocriptine prior to pregnancy or in the early stages generally experienced favorable pregnancy outcomes, consistent with findings in existing literature (Molitch, 2015; Turkalj et al., 1982; Huang and Molitch, 2019). However, Group A, which discontinued bromocriptine 1 month prior to pregnancy and did not receive any treatment during pregnancy, showed a higher incidence of neurological symptoms, such as headaches and vision disturbances, compared to Groups C and D. This aligns with previous studies suggesting that pregnancy-induced hormonal changes, particularly elevated estrogen levels, may exacerbate tumor growth in untreated or suboptimally managed prolactinomas. Our findings further suggest that, for patients with neurological or ophthalmological symptoms before pregnancy, there is an elevated risk of tumor progression during gestation (Maiter, 2016). Two patients in Group A required pregnancy termination due to rapid symptom deterioration and underwent neurosurgical intervention, highlighting the importance of careful monitoring and individualized management in these cases.

Regular monitoring during pregnancy is essential, particularly for those with pre-existing neurological symptoms or untreated adenomas. While routine visual field exams may not be necessary for all patients, current guidelines recommend close monitoring for those at higher risk of tumor growth, with visual field exams performed every 2 months. If symptoms suggest tumor progression, cranial MRI can be used to confirm the diagnosis, and DA therapy can be reintroduced based on symptom severity and gestational age. In extreme cases, surgical intervention may be required (Gemzell and Wang, 1979).

Secondly, the impact of bromocriptine on fetal outcomes remains a topic of ongoing investigation. In our study, the incidence of fetal malformations was 1.6%, which is consistent with previous reports on bromocriptine use during pregnancy (Krupp and Monka, 1987), below the total incidence rate of birth defects in China, which is 5.6% (Xie et al., 2018). We observed a trend toward an increase in small-for-gestational-age (SGA) infants in patients with prolonged bromocriptine use, particularly in Group D, but this trend was not statistically significant, which demonstrates the safety of DA during pregnancy. But the potential impact of long-term bromocriptine usage on fetal development, particularly with regard to growth restriction, requires further larger cohorts and prospective designs to clarify the long-term impact on fetal health.

While the mode of delivery varied across the groups, with Group D having the highest cesarean section rate (77.5%), this difference may be attributed to the higher incidence of adverse obstetric history and assisted reproduction in this group rather than a direct effect of bromocriptine use. Moreover, we found no significant differences in the rates of gestational diabetes or pregnancy-induced hypertension between the groups, further reinforcing the safety of bromocriptine with respect to maternal health.

Current international guidelines generally recommend discontinuing bromocriptine upon pregnancy confirmation to minimize fetal exposure (Petersenn et al., 2023). However, cabergoline, another dopamine agonist with a longer half-life and greater efficacy, has been increasingly considered for use in pregnancy (Barraud et al., 2020). While fewer studies have evaluated cabergoline (Sant' Anna et al., 2020; Hurault-Delarue et al., 2014), emerging evidence suggests it may be as safe as bromocriptine in terms of pregnancy outcomes (Krupp and Monka, 1987; Huang and Molitch, 2019). The latest consensus emphasizes the need for individualized treatment, particularly in women with larger or more invasive prolactinomas (Petersenn et al., 2023). Although this study focuses on bromocriptine, future research comparing the safety and efficacy of bromocriptine and cabergoline during pregnancy is needed, especially given cabergoline’s growing clinical use.

### 4.1 Limitations

This study has several limitations. As a retrospective, single-center study, there is a potential for selection bias, which may limit the generalizability of the findings. Our study primarily focuses on patients with prolactin-secreting microadenomas in the second and third trimesters, and does not include patients who experienced miscarriages in the early stages of pregnancy. Future studies should include all patients with pregnancy-associated prolactin-secreting microadenomas. Dose-response analysis was not conducted due to the variety of inadequate details on bromocriptine exposure,and the lack of randomization causes differences in baseline characteristics. The absence of pre-existing comorbidity data is another limitation of our study.

Additionally, while our study included detailed pregnancy and neonatal outcomes, we did not assess changes in prolactinoma size during pregnancy. Future studies should incorporate regular monitoring of tumor size to provide a more comprehensive understanding of the impact of pregnancy on prolactinomas.

In addition, prospective, multicenter studies comparing different dopamine agonists, including cabergoline are needed to guide clinical practice more effectively.

## 5 Conclusion

In conclusion, our study supports the safety of discontinuing bromocriptine in early pregnancy for most women with prolactin-secreting microadenomas. However, for patients with pre-existing neurological symptoms or untreated prolactinomas, the risk of tumor progression during pregnancy may necessitate continued DA therapy and close monitoring. Our study indicates continuing DA in pregnancy did not increase miscarriage or fetal anomaly rates or adverse neonatal outcomes. but the potential impact of long-term bromocriptine usage on fetal development, especially in terms of fetal growth warrants further prospective research. Moving forward, individualized treatment plans, guided by the severity of the prolactinoma and maternal health status, will be essential to optimizing both maternal and fetal outcomes.

## Data Availability

The original contributions presented in the study are included in the article/supplementary material, further inquiries can be directed to the corresponding authors.
